# COVID-19 Surge Capacity Solutions: Our Experience of Converting a Concert Hall into a Temporary Hospital for Mild and Moderate COVID-19 Patients

**DOI:** 10.1017/dmp.2020.412

**Published:** 2020-10-26

**Authors:** Daniela Sacchetto, Mario Raviolo, Cristiano Beltrando, Nicola Tommasoni

**Affiliations:** Disaster Medicine Service 118, ASL CN1, Saluzzo, Italy

**Keywords:** COVID-19, pandemic, temporary hospital, mild moderate patients

## Abstract

The 2019 coronavirus disease (COVID-19) pandemic has stressed the health care system in Italy as well as around the world, with hospitals implementing their surge capacity to increase the number of available beds for patients positive to the virus.

At the end of March, the Piemonte (Northern Italy) Government decided to build a temporary rapid-assembly emergency hospital for the treatment of mild and moderate COVID-19 patients, converting an existing concert hall in the city of Turin. The decision was prompted not only by the urgent need of hospital beds, but also by a forward-looking approach for the months immediately after the emergency, when it will be essential for conventional hospitals to return to a normal configuration.

This paper documents the temporary hospital project, describing the site, the layout and the equipment, the idea behind structural choices, and the staff involved. The aim of the work is to share the experience and to provide some practical recommendations to other professionals who are fighting the COVID-19 pandemic worldwide.

The 2019 coronavirus disease (COVID-19) began in China in early December 2019 and rapidly spread to many countries around the globe. The World Health Organization (WHO) defined the emergency as a pandemic at the beginning of March 2020.^1^

In Italy, the emergency started on February 20, 2020, when the first case of COVID-19 was registered in Codogno, Lodi Province, Lombardia, Northern Italy. In the following days, the disease cases were found in other regions of Northern Italy, including Piemonte. Starting at the end of February 2020, the number of patients affected with COVID-19 registered in Piemonte began to increase exponentially, stressing the Regional Health System with hospitals challenging to cope with the surge of patients affected by COVID-19: the hospitals implemented their surge capacity, modifying the configuration of their wards and intensive care units (ICUs), to increase the number of available beds for COVID-19-positive patients. In particular, the number of ICU beds available in Piemonte was increased from 287 to 554, and the number of “sub-intensive” beds was increased from 90 to 270.^2,3^

Under these circumstances, on March 27, 2020, the Piemonte Government evaluated the idea of building a rapid-assembly emergency hospital for the treatment of mild and moderate COVID-19 patients. Although the request of hospital beds for COVID-19 patients was slightly decreasing at the end of March, this decision was prompted not only by the urgent needs, but also by a forward-looking approach: during the months immediately after the emergency, it will be important to have a place in which it will be possible to admit COVID-19 patients, allowing the conventional hospitals to clean and set up the wards for common inpatients.

The final temporary hospital consisted of a total of 90 beds, organized as 4 ICU beds, 30 “sub-intensive” beds, and 56 ward beds. The setup works started on April 4, and the first patient was admitted on April 19.

The aim of this paper is to document the conception and building of a temporary hospital during the COVID-19 pandemic in Northern Italy, reporting our experience in the project from the first idea, as recommendations and information for other countries and professionals who are fighting the emergency worldwide.

## Narrative

### Site and Idea

The place identified to set up the temporary hospital was the concert hall at Officine Grandi Riparazioni (OGR), in Turin, which will be referred to as *OGR Temporary Hospital* in the following. The site presented numerous logistics advantages, such as the availability of about 6000 sq m, covered and heated, the availability of a high power electricity system (due to normal use for concerts), and the availability of a parking space for personnel. Moreover, a fundamental strong point that the site demonstrated was the presence of 2 separated air treatment units that allowed a light negative pressure area where identified COVID-19 patients will be admitted (red-zone).

The conversion of the site from a public show place to a COVID-19 temporary hospital required the development of oxygen and electricity systems, the building of temporary walls, and the installation of toilets and showers (pre-assembled containers) to increase the number of bathrooms available for patients and workers.

Following a criterion of territorial relevance, the Piemonte Government decided to set up this temporary hospital as a virtual extension of the Azienda Sanitaria Locale Città di Torino (ASLTO) that, since the building phase was finished, manages all the running operations as an additional and displaced ward of one of its conventional hospitals.

The target of this temporary hospital was to accommodate mild and moderate COVID-19 patients: if their conditions become more severe, they will be transferred to a higher-level traditional hospital for further treatment. The ICU beds were designed and implemented only to stabilize patients while waiting for referral to the identified conventional hospital.

### Layout and Equipment

**Figure 1.**
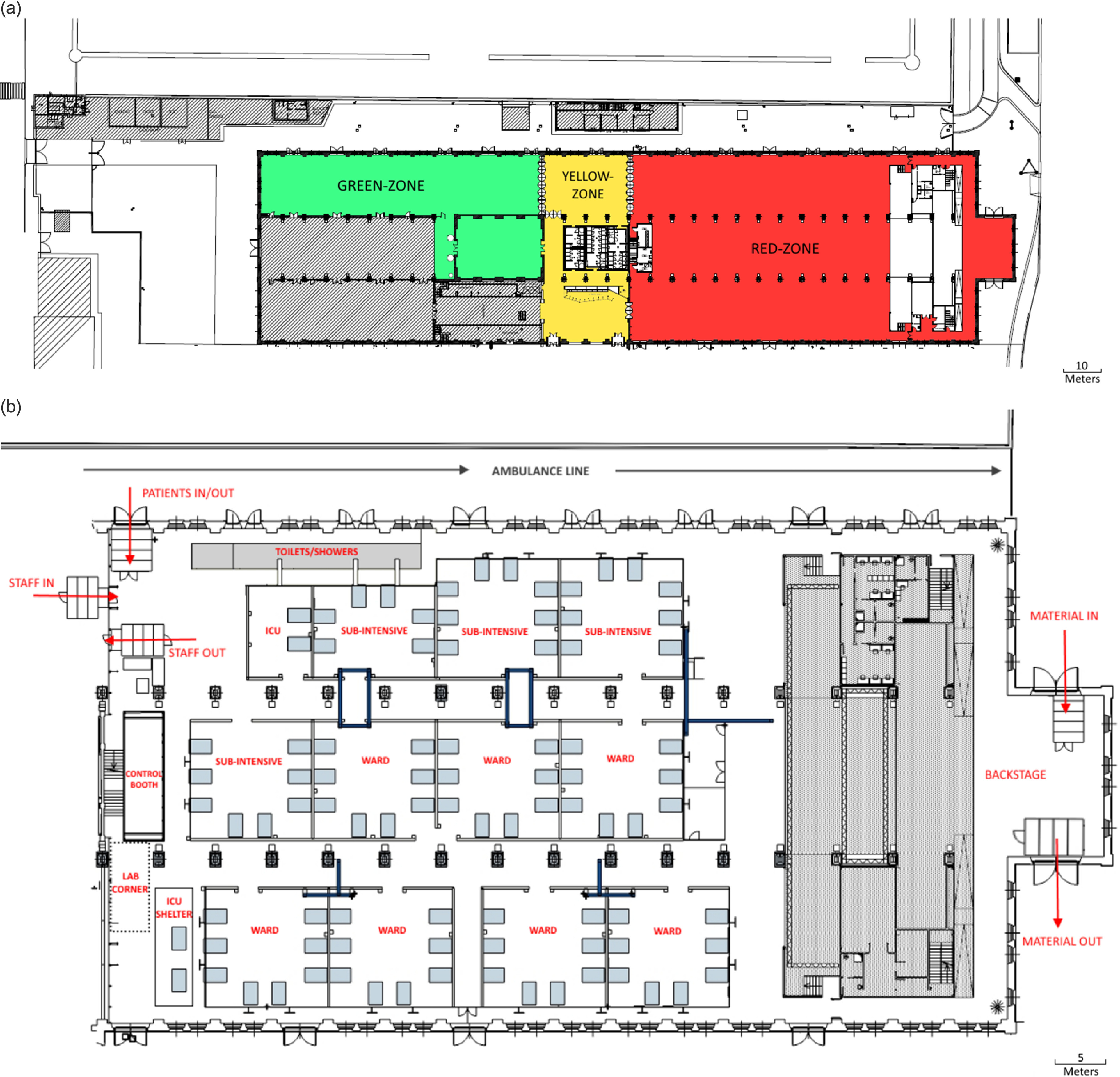
(a) The OGR Temporary Hospital was divided into 3 areas: red-zone, a contaminated area with COVID-19 patients; yellow-zone, an intermediate area between clean and contaminated ones; and green-zone, a clean area for managing activities not related to patients. Shaded areas stand for sectors not available for the hospital. (b) Red-zone layout of the OGR Temporary Hospital.

Figure 1a shows the site, divided into the 3 main areas: (a) the red-zone, a contaminated area in which patients are admitted and personnel can enter only wearing adequate personal protective equipment (PPE); (b) the yellow-zone, a semi-clean area where the health workers put on the PPE; and (c) the green-zone, a clean area in which personnel can work as usual, without PPE and in which there is a clean warehouse for expensive material that is safer to not keep in the red-zone, such as drugs or backup devices.

Figure 1b details the red-zone. The 90 beds were organized in 12 boxes and 1 shelter, separated by 2 main aisles; the boxes, containing 2, 6, or 8 beds each, were designed to assure different levels of care (the smaller ones are for ICU beds) and reasonable privacy for patients, in addition to being a valid support to build the electrical and oxygen systems. In particular, the separating walls of boxes were designed 1.5 m tall; the idea was to assure both privacy and reduction of personnel needed to take care patients. In fact, separated rooms as in a conventional hospital, force a higher health care workers to patients ratio because health care providers are unable to see inside the other rooms compared to a “semi-open” space.

Two specific areas of the concert hall were used taking advantage of their position and their characteristics: the backstage and the control booth. The *backstage* was designed to accommodate an area for the unload and re-supply of materials that can be stored in the red-zone, and an exit line for the dirty materials (linens and clothes, waste, bodies, etc). These routes were easily accessible from a main road and were separate from both the ambulance line and patient entrance and discharge. The *control booth* was set up as the direction and coordination room: this space was on an upper level compared to the patients’ area, allowing the medical director and the nurse coordinator to have a global overlook on the red-zone.

All the ways to enter in and to exit from the red-zone for personnel, patients, and materials were protected by passages; each of these passages was designed as a small room closed with a ceiling, provided with 1 window to see inside before entering and with a swing door in the side closest to the red-zone.

The number of available toilets and showers in the red-zone was 18 and 8, respectively, increasing the building’s normal dotation with 4 shelters located along the first line of boxes. Two additional sinks, dedicated to health workers’ necessities, were installed at the 2 extremities of the first aisle.

Regarding the oxygen supply system, the decision taken for the OGR Temporary Hospital was to use Oxygen 93 manufactured on-site, using an oxygen pressure swing adsorption (PSA) concentrator unit able to produce 60 m^3^/hr of gas. PSA concentrators are normally used in field hospitals by military forces and are listed as a solution in the WHO Interim Guidance regarding the oxygen sources and strategies for COVID-19 treatment.^4^

Concerning the electrical supply system, all the 90 patients’ spaces were equipped in the same way to assure flexibility and transformability in case of further needs. In particular, in addition to the oxygen supply point, each bed was equipped with 6 electrical universal plugs, 1 lamp, 1 service plug, and 1 patient alarm. A backup generator of about 600 kW power was located outside the building to be used in case of failure of the primary electrical supply.

From a safety point of view, the installation of 1 ceiling video camera for each box and for the aisle along the toilets shelters allows the supervision of the patients’ situation from the direction/coordination room.

Finally, the entire red-zone was wired to allow a local area network connection for the ICU and “sub-intensive” beds, whereas a Wi-Fi network was established to allow the use of electronic clinical patient records inside the OGR Temporary Hospital.

Regarding the medical devices, “sub-intensive” beds are equipped with a monitor, continuous positive airway pressure system, suctioning system, and syringe pumps rack, whereas the ICU beds include a ventilator in addition to all the previously listed equipment. Among others, 2 defibrillators, 4 ultrasound machines, 2 electrocardiographs, 2 scialytic lamps, and 2 refrigerators for drugs were positioned along the 2 main aisles. In a dedicated corner, some laboratory machines for routine blood tests and 1 X-ray mobile unit for chest exams were allocated to be easily used in case of need.

### Staff

The ASLTO team served as the leading clinical team, recruiting anesthesiologists, emergency doctors, nurses, and other health care professionals, such as laboratory and X-ray technicians, to work in the OGR Temporary Hospital. In addition to these personnel, a team of 37 health care professionals came from Cuba^5^ to help Piemonte face the COVID-19 pandemic.

## Discussion

As the recent literature^6-12^ and news around the world^13-15^ show, the idea to convert a public place into a temporary hospital has been widely used during the COVID-19 emergency, especially in China, with the opening of 16 “Ark/Fangcang” shelter hospitals in Wuhan, treating more than 12 000 patients in stadiums and exhibition centers. In fact, temporary hospitals can give an immediate response to the request of new beds from stressed hospitals, with time and cost reduction compared to the building of new conventional hospitals or conventional wards.

However, the solution implemented with OGR Temporary Hospital brings along some important considerations. First, the Piemonte idea was different both from what was done in China with shelter hospitals and from what was done in Lombardia^15^: the point of strength of this solution is to have a potentially replicable model both to cope with the need of beds by stressed hospitals and to admit future COVID-19 patients while conventional hospitals restore to the pre-pandemic configuration. Moreover, building a temporary hospital targeted for only mild and moderate patients allows not only to contain costs regarding equipment and staff, but also to avoid the risk of the temporary hospital being ready, since the building phase lasts at least 10–15 days from the decision to build it, when the peak of severe and critical patients is already decreasing, but the need of lower level beds is still ongoing.

Second, the implemented “semi-open” space layout, together with the type of patients admitted, allowed for a reduction of the amount of personnel involved, which is a considerable problem in the case of such a contagious disease, stressing the health system both for the effort required by health workers and for the number of health workers getting sick every day.

Finally, to avoid what is reported by Yuan et al.^8^ about patients feeling worried and marginalized by the admission into nonconventional hospitals, the OGR Temporary Hospital was built assuring high-standard clinical devices, high technology, nice furniture, and attention to structural details. These important elements helped the patients feel as though they were being treated the same way as patients in a conventional hospital.

## Conclusion

It is clear that this paper has a time-dependent limitation for now, due to lack of data about patients admitted and team working dynamics; nevertheless, the urgent need for information pushed us to share our experience, in a moment in which all available data can be useful to better fight the problem.
